# STATE LEADERS DESCRIBE EXCITING AND REPLICABLE AGE-FRIENDLY WORK

**DOI:** 10.1093/geroni/igae098.0302

**Published:** 2024-12-31

**Authors:** Nina Silverstein

**Affiliations:** University of Massachusetts Boston, Needham, Massachusetts, United States

## Abstract

State leaders describe age-friendly work in their state and how tools like the healthyagingdatareports.org can be leveraged to expand and accelerate progress. James Fuccione (Massachusetts), Jennifer Rabalais (New Hampshire), Kina White (Mississippi), and Virginia Vincenti (Wyoming) will provide insights about their state. Silverstein will identify common themes and lessons learned.

